# Preprosthetic Management of “Flabby Ridge” on Edentulous Patient

**DOI:** 10.1155/2021/6613628

**Published:** 2021-03-16

**Authors:** Amani Mizouri, Oumaima Tayari, AlaEddine Mahfoudhi, Adel Bouguezzi, Jamila Jaouadi

**Affiliations:** ^1^Complete Removable Prosthodontic Department, Research Laboratory LR12ES11, Faculty of Dental Medicine, University of Monastir, Monastir, Tunisia; ^2^Oral Surgery Department, Research Laboratory LR12ES11, Faculty of Dental Medicine, University of Monastir, Monastir, Tunisia

## Abstract

For edentulous patients, the integrity of the osteomucosal-bearing surface is a significant factor for the balance of the future removable prosthesis. The supporting tissues are influenced by several factors. Physiological bone resorption, senescence, and changes caused by systemic diseases and polypharmacy lead to modifications in these tissues. Similarly, trauma caused by an ill-fitted prosthesis influences the degree of bone resorption and the development of hyperplastic tissue. The etiology of bone resorption is multifactorial and complex, with continuous individual variations that are often unexplained. Although, no study has been able to establish the importance of the different factors in relation to each other, it is often reported that chronic excessive mechanical pressure in relation to occlusal constraints is responsible for the appearance of local resorption's areas. The aim of this work is to present, through three clinical cases, the various factors involved in the alteration of the osteomucous-bearing surface and eventually, possible therapeutic attitude to deal with.

## 1. Introduction

The use of an ill-fitting or very old complete prosthesis leads to deep changes of the ecosystem, in the oral cavity, and can cause tissue damage [1].

Among the many injuries related to wearing a misadjusted removable prosthesis, prosthetic stomatitis, angular cheilitis, disinserted fibromucosa or “floating ridge,” and fibrous hyperplastic lesion are included [2, 3].

An impression taken on altered or deformed tissue usually leads to the realization of an unstable prosthesis which will accelerate bone resorption.

This is why restoring a healthy state of osteomucous support appears as an indispensable preliminary condition for a consistent and efficient prosthetic treatment [3].

The success of prosthodontic treatment in edentulous patients depends closely on the state of the osteomucous surface that supports the prosthesis and requires a quantitative and qualitative evaluation of the bone and mucosal components [3, 4].

For the bone support, several parameters are evaluated, such as the degree of resorption: in fact, a residual bone represents a favorable factor to the prosthetic balance.

In case of significant resorption compromising the prosthetic retention, treatment with implant can be envisaged after a preimplant assessment [4].

Mucous membrane, due to its anatomical and histological particularities, can interfere with the use of prosthesis. Mucosal lesions induced by the complete prosthesis can be classified according to their etiology in the following:

- Traumatic injuries, especially anterior occlusal overload, caused by a defective and traumatic complete prosthesis for the underlying osteomucous-bearing surfaces, such as the case of flabby ridges and epulis fissuratum [1, 3, 5–8]
- Prosthetic stomatitis which is defined as a chronic inflammatory condition of the oral mucosa supporting a removable prosthesis. Its etiology is multifactorial, mainly, the trauma caused by ill-fitting prostheses associated to an infectious factor, oral and prosthetic deficient hygiene, continuous prosthetic use, salivary dysfunction, and smoking [1, 4, 9–11]

Management of prosthesis inducing mucosal lesions depends on the type of the lesion and its etiology. The treatment consists of several phases, either separately or concomitantly: tissue conditioning, special impression techniques for compromised ridges, and surgical option.

The purpose of this paper is to present various preprosthetic management techniques of hyperplastic soft tissue on edentulous patient.

## 2. Case Report 1

A 60-year-old man, with no medical history, was referred to the department of prosthodontics of the faculty of dental medicine of Monastir, Tunisia, requesting a new set of complete prosthesis.

**Figure 1:**
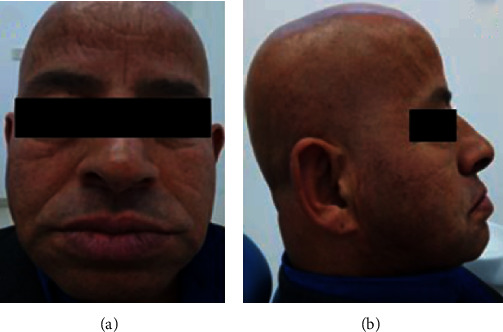
Extraoral appearance: (a) front view and (b) side view.

The extraoral examination revealed the decrease of occlusal vertical dimension (OVD) (Figures 1(a) and 1(b)).

**Figure 2:**
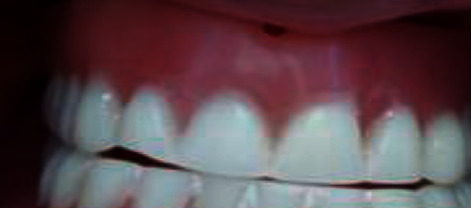
Prosthesis examination.

An intraoral examination allowed the evaluation of the existing prosthesis for which the occlusal plane was correctly oriented in the maxillary prosthesis obeying to esthetic and phonetic guidelines; the centric relation (CR) was faulty: the patient was used to make a protrusive occlusion so it was so difficult to guide him in correct CR because of his acquired occlusal memory, and the border extensions of maxillary and mandibular complete prostheses were correctly situated (Figure 2).

The maxillary and mandibular edentulous ridges showed a significant bone resorption, covered by inflamed fibromucosa.

**Figure 3:**
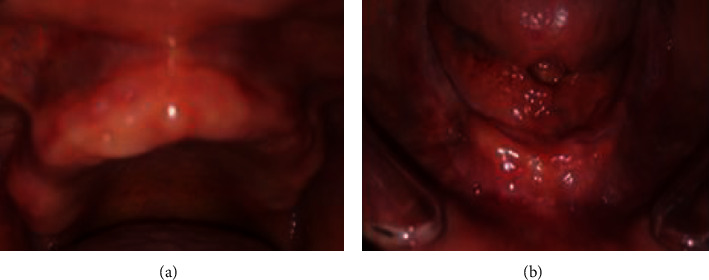
Intraoral examination: (a) inflamed maxillary fibromucosa and (b) inflamed and flabby tissue in the mandibular anterior region.

A flabby tissue in the anterior mandibular ridge extending from the canine to canine region was noticed (Figures 3(a) and 3(b)).

According to the data obtained, a diagnosis of a flabby ridge of the anterior mandibular region and a generalized prosthetic stomatitis due to the excessive and continuous mechanical pressure imparted by the faulty prosthesis's occlusion were performed.

A new complete prosthesis was planned for this patient concomitantly with an occlusal conditioning treatment followed by a vestibuloplasty to restore anterior alveolar ridge height.

The patient was informed in detail about the procedure, and his consent was obtained.

At the first appointment, a modification of the mandibular prosthesis using self-cured acrylic resin over the artificial teeth served to restore the vertical dimension and centric relation provisionally until fabricating new prostheses in order to break up with old protrusive reflex.

The procedure was to wrap two strands of wax and place them over teeth occlusal surfaces and record the new mandibular position (TENCH articulating); the purpose of this step is to guide the patient to close in CR that has been validated by finding the coincidence of the interincisal midpoint.

**Figure 4:**
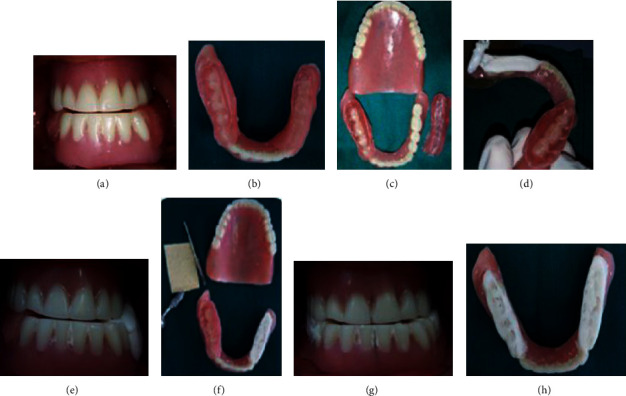
TENCH articulated technique. (a, b) Interocclusal record using wax. (c, d) Replacing the wax with chemopolymerized resin on the right side. (e) Centric relation. (f) Prosthesis polishing. (g, h) Procedure on the right side.

Then, the wax on the left side was replaced with self-cured acrylic resin and the same operation was repeated. Finally, eliminate the excess of resin, polish the prostheses, and recheck accuracy of previously made centric relation record (Figures 4(a)–4(h)). The same procedure was repeated on the right side.

This new occlusal situation will serve as a guide for manufacturing new complete dentures.

**Figure 5:**
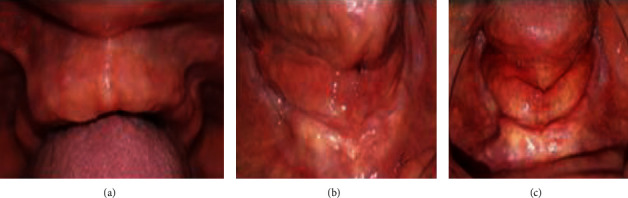
Regression of the inflammation of the maxillary and mandibular fibromucosa after occlusal conditioning treatment: (a, b) one week and (c) two weeks.

The fibromucosa status was evaluated in one and 2 weeks after the occlusal conditioning treatment; there was a significant regression of the inflammation (Figures 5(a)–5(c)). However, there was a persistent hyperplastic ridge that established an unfavorable bearing surface for the new prosthesis. During the next appointments, the clinical procedures for the new complete prosthesis were initiated following the conventional technique.

After manufacturing the final prostheses, surgical procedures were planned starting with vestibuloplasty, followed by placing an epithelioconnective palatal flap.

For this, an infiltrative local anesthesia technique (2% lidocaine) was necessary before performing soft tissue surgery. A deep incision was carried from the canine to canine region on the labial surface of the mandibular arch; then, the vestibule was deepened by a supraperiosteal dissection. The mucosal flap was turned downward from its attachment and placed directly against the periosteum to which it was sutured with interrupted ones.

**Figure 6:**
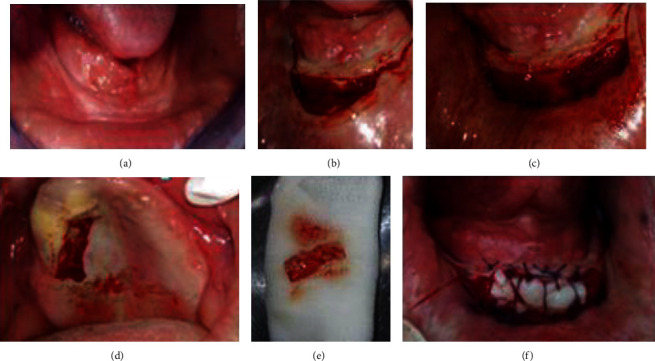
The surgical procedure: (a) preoperative view, (b) horizontal semilunar incision followed by Supra periosteal dissection deepening the vestibule, (c) suturing of the mucosal flap, (d, e) epithelioconnective palatal flap, and (f) immediate postoperative view.

The newly exposed periosteum was covered by the epithelial-connective tissue graft using a palatal flap (Figures 6(a)–6(f)).

Postsurgical prosthesis withdrawal was beneficial to not irritate the graft.

Antibiotics (amoxicillin 2 g per day for 7days), an anti-inflammatory drug (ibuprofen 600 mg every eight hours for 4 days), an analgesic (Paracetamol 650 mg every eight hours for 4days), and 0.12% chlorhexidine mouthwashes (used from the second day postsurgery) were prescribed.

**Figure 7:**
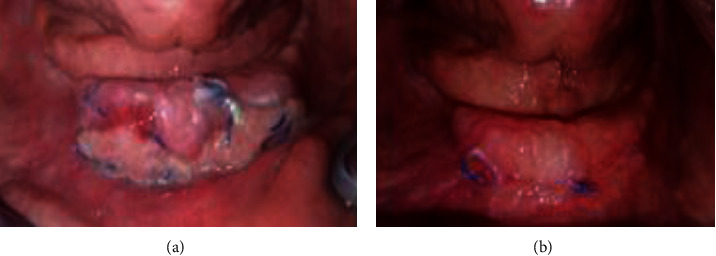
Healing process of the treated area: (a) 7 days and (b) 14 days.

The patient was recalled for follow-up (7 days and 14 days) (Figures 7(a) and 7(b)).

**Figure 8:**
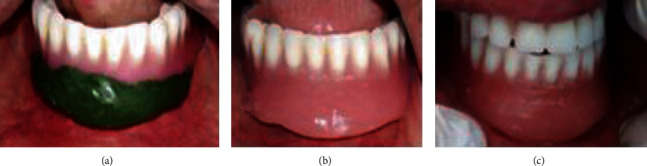
Prostheses adjustments: (a) border molded mandibular prosthesis, (b) chemopolymerized resin molding, and (c) occlusal adjustments.

Once healing was complete, the anterior edge of the mandibular prosthesis must be adjusted to the new vestibular height. The lack was adjusted by a border-molded impression compound that was replaced later with chemopolymerized resin. Finally, occlusal adjustments were performed (Figures 8(a)–8(c)).

**Figure 9:**
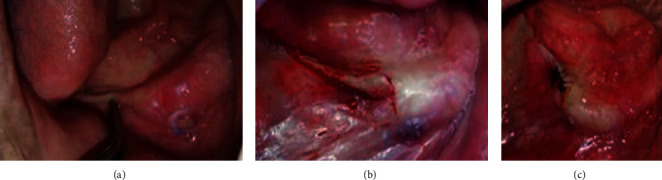
Surgical correction of the disinserted fibromucosa: (a) clinical examination of the disinserted fibromucosa and (b, c) surgical treatment.

In addition, a surgical correction of the disinserted fibromucosa at the posteromandibular ridge is necessary and managed by a surgical correction which includes simple trimming of the excessive tissue but not disturbing the attached fibromucosa (Figures 9(a)–9(c)).

## 3. Case Report 2

A 64-year-old-male patient consulted the prosthodontic department of the faculty of dental medicine of Monastir requesting new prostheses. His complaints were mainly about esthetic appearance and ill-fitting prostheses causing discomfort while it is used. He had been wearing the current prostheses for more than 10 years.

**Figure 10:**
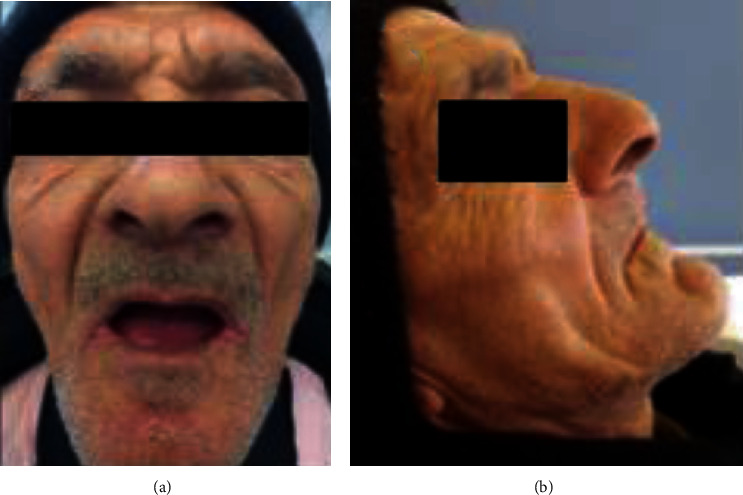
Extraoral examination: (a) front view and (b) side view.

Extraoral examination revealed a decrease of the occlusal vertical dimension which caused angular cheilitis (Figures 10(a) and 10(b)).

**Figure 11:**
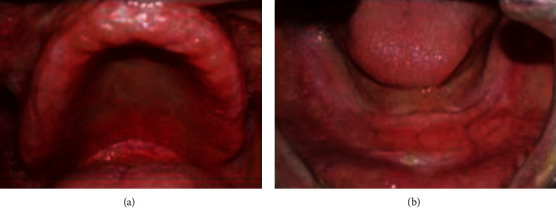
Intraoral examination: (a) flabby tissue in the maxillary anterior region and (b) fibrous hyperplastic mass.

Intraoral examination showed edentulous maxillary and mandibular resorbed ridges covered with an inflamed fibromucosa. An area of flabby tissue in the maxillary anterior region and a fibrous hyperplastic mass in relation with the anterior mandibular vestibule were found (Figures 11(a) and 11(b)).

Both maxillary and mandibular covering tissues presented an extensive inflammatory lesion due to traumatic occlusion and prosthetic stomatitis confirmed by oral mycological analysis. A preliminary tissue conditioning treatment associated to antifungal agent (0.2% chlorhexidine mouthwash) was indispensable.

**Figure 12:**
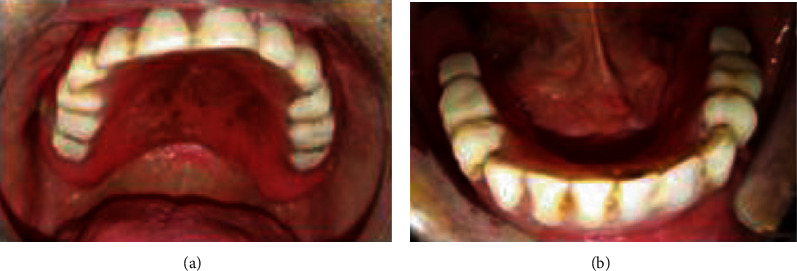
Prosthesis examination: (a) old maxillary prothesis and (b) old mandibular prosthesis.

**Figure 13:**
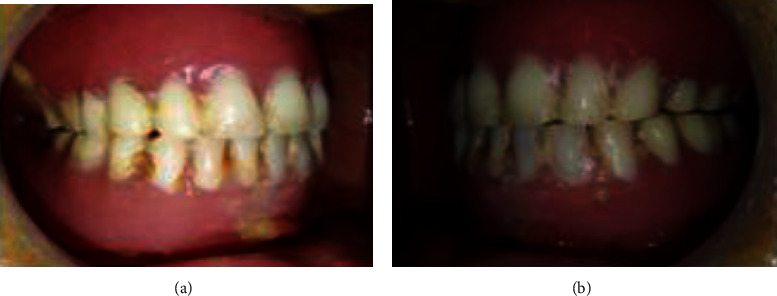
Misadjusted occlusion: (a) right side and (b) left side.

Prosthesis examination revealed teeth abrasion, insufficient prosthesis hygiene, and misadjusted occlusion causing trauma to the area during swallowing and mastication (Figures 12 and 13).

A clinical diagnosis of an epulis fissuratum due to chronic irritation from the ill-fitting prosthesis was evoked. The oral patient consent was obtained in order to fabricate a new complete prosthesis followed by a surgical excision of the lesion and vestibuloplasty to avoid recurrence.

**Figure 14:**
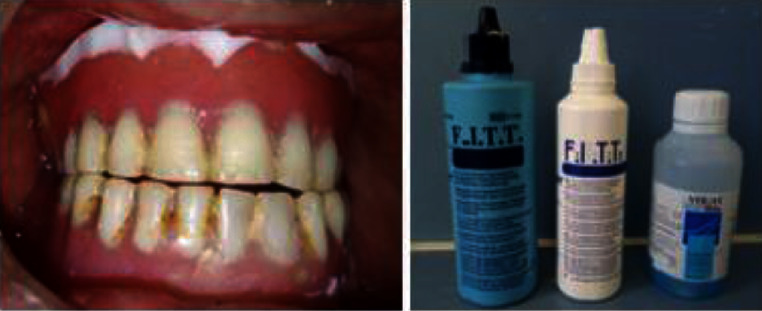
Tissue conditioning using soft tissue conditioner mixed with an anti-inflammatory mouthwash.

**Figure 15:**
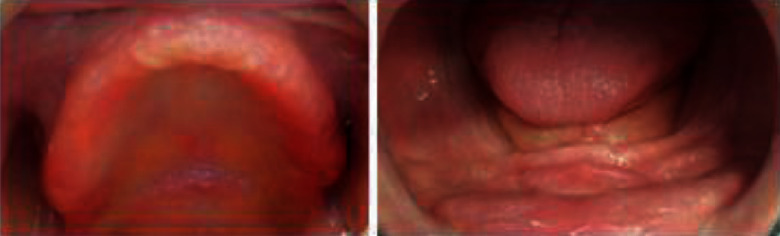
Healthy oral fibromucosa further to a tissue conditioning treatment.

At the first appointment, the patient's existing prostheses were evaluated for fit and occlusion. Then, prostheses were relined according to the present residual ridges using the temporary soft resin (FITT Kerr®) to improve the state of the soft tissues before manufacturing new prostheses. The procedure is repeated every 2-3 weeks until the soft tissues recovery (Figures 14 and 15).

The patient is now ready for the fabrication of new prostheses.

**Figure 16:**
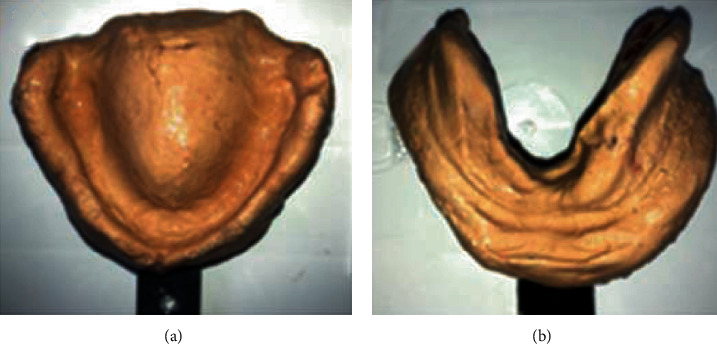
(a) Preliminary impression. (b) The epulis fissuratum area's delineation on the mandibular preliminary impression.

The preliminary impressions were made using impression plaster to record hyperplastic tissues in the uncompressed state (Figures 16(a) and 16(b)). This negative record was meticulously taken so that the compression was minimized; no major pressure spots were found. The impression was poured using a white dental plaster.

**Figure 17:**
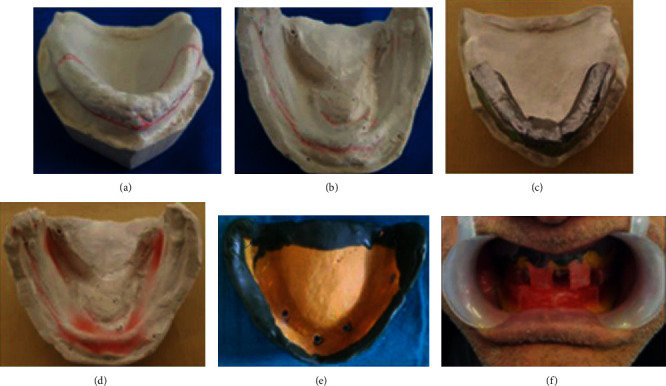
Prerequisites to the final impression. (a, b) The hyperplastic maxillary and mandibular area delineation scribed on the preliminary cast. (c) Dental tin foil over the maxillary hyperplastic area. (d) Blocking out the undercut as well as redundant tissue on the cast with wax. (e) Perforations corresponding to the hyperplastic area. (f) Final impression under occlusal pressure.

The floating tissue is perfectly defined by palpation and scribed on the cast (Figures 17(a) and 17(b)). Then, it was blocked out by applying a 3 mm thick layer of dental tin foil over the maxillary hyperplastic area (Figure 17(c)). The special tray was made using a self-curing resin. Two occlusion rims were contoured and adjusted to record centric relation at the adequate occlusal vertical dimension. Border molding was done using a green stick compound (Kerr Impression Compound Green®). After testing the retention, holes were drilled into the special tray, in front of hyperplastic area, with a round bur to remove the excess of impression material. The final impression was taken with Permalastic Polysulfide Impression Material, Kerr Dental®, using the functional pressure; the patient was gently guided to centric relation at the correct vertical occlusal dimension until the material setting (Figures 17(d)–17(f)).

**Figure 18:**
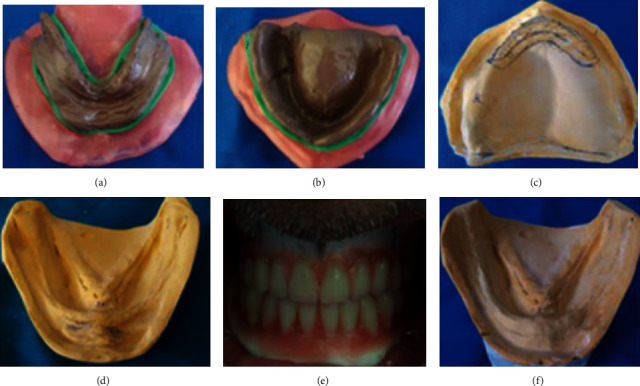
(a, b) Boxing impression. (c, d) The secondary casts. (e) Teeth arrangement try-in. (f) Surgery simulation.

Final impressions were boxed and poured to get final casts on which record bases were fabricated and occlusion rims were prepared (Figures 18(a)–18(d)).

The occlusal plane was oriented using esthetic, phonetic, and anatomic guidelines. Then, occlusal vertical dimension was established using the physiological rest position associated with phonetic and esthetic techniques. Finally, the centric relation recording was done followed by the transfer of jaw relation to a semiadjustable articulator. Maxillary cast was mounted using facebow record, while mandibular cast was mounted using interocclusal record.

During the try-in appointment, the occlusion and the aesthetic integration of the prosthetic reconstruction were verified and accomplished (Figure 18(e)).

Before manufacturing the final prostheses, surgical procedures must be simulated on the final cast by scarping the amount of plaster corresponding to the ulterior removed tissue (Figure 18(f)).

**Figure 19:**
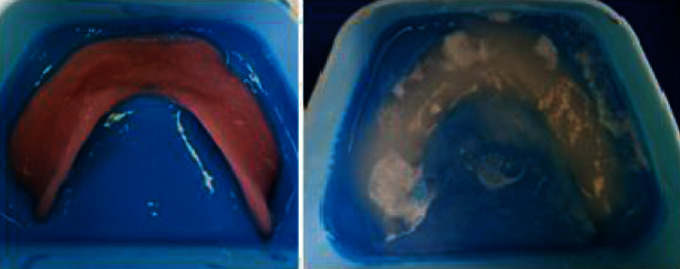
Prosthesis duplication.

After manufacturing, the final mandibular prosthesis must be duplicated with transparent resin which served as a surgical guide (Figure 19).

**Figure 20:**
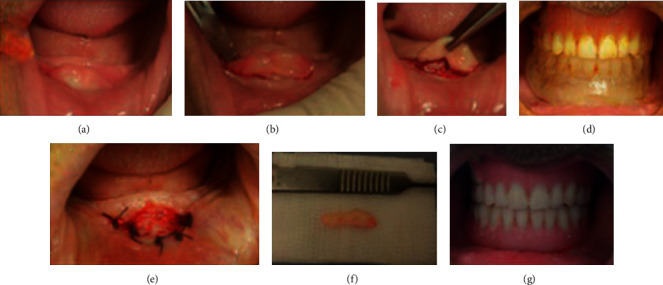
Surgical procedure: (a) infiltrative local anesthesia, (b) semilunar incision, (c) total excision using conventional scalpel surgery, (d) surgery control using surgical guide, (e) immediate aspect of the treated area: suture preserving the depth of the buccal side in the mandible. (f) operatory specimen of the mandibular lesion. and (g) frontal view after immediately prostheses insertion and occlusal adjustment.

The excision of the epulis fissuratum was performed with the conventional surgical procedure that was controlled by the surgical guide until obtaining the wanted width of the vestibule (Figures 20(a)–20(d)).

The depth of the vestibule was preserved. Bleeding control by compression and primary closure was performed with interrupted sutures (Figure 20(e)). All specimens obtained from the patient were sent for histopathological examination to confirm the diagnosis.

Healing was done by secondary intention (Figures 20(f) and 20(g)).

**Figure 21:**
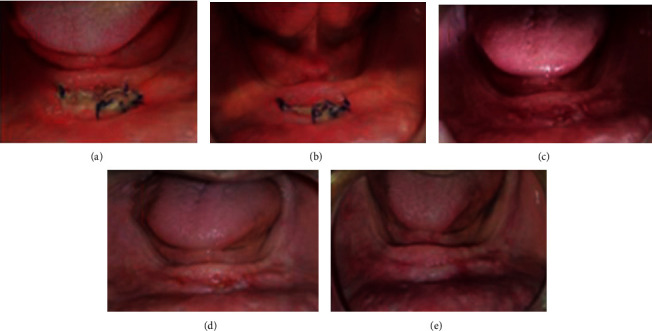
Healing process of the treated area: (a) 3 days, (b) 1 week, (c) 2 weeks, (d) 3 weeks, and (e) 4 weeks.

Minor occlusal adjustments were performed immediately after surgery and prostheses should be kept in place continuously during the first 2-3 days. Patient was recommended to start rinsing with a chlorhexidine mouthwash, the second day after surgery, three times a day during the postoperative first week. Amoxicillin and paracetamol were prescribed. Postoperative controls were performed 3 days and 1, 2, 3, and 4 weeks for the assessment of wound healing (Figures 21(a)–21(e)).

## 4. Case Report 3

A 57-year-old hypertensive male reported to the prosthodontic department with the chief complaint of an ill-fitting of his complete prostheses delivered about 7 years ago.

In addition, patient complained of pain and discomfort during mastication.

**Figure 22:**
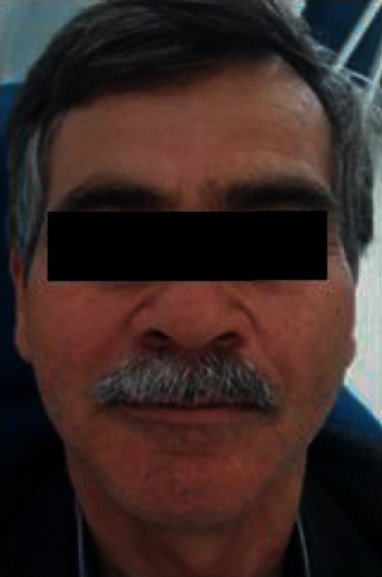
Extraoral view.

Extraoral examination revealed a decrease in the occlusal vertical dimension (Figure 22).

**Figure 23:**
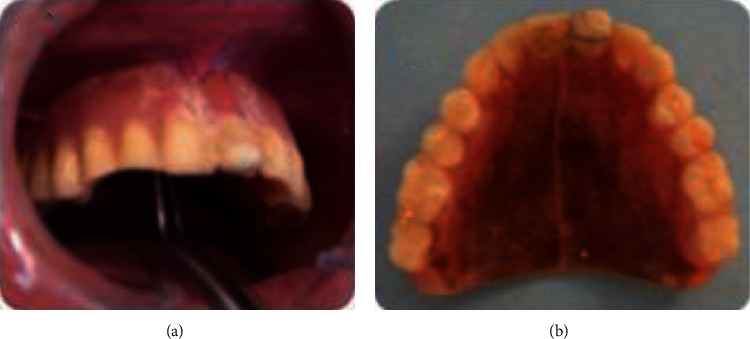
(a, b) Prosthesis examination: an ill-fitting prostheses.

The clinical exam showed that the upper prosthesis was ill-fitting because of its multiple reparations and teeth replacement (Figures 23(a) and 23(b)).

**Figure 24:**
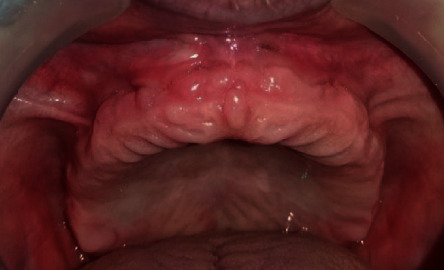
Intraoral view: right lateral frenulum.

On visual inspection, a fibrous hyperplastic mass in relation with the anterior maxillary vestibule was noticed. It extended to the anterior crest of the residual ridge (Figure 24).

**Figure 25:**
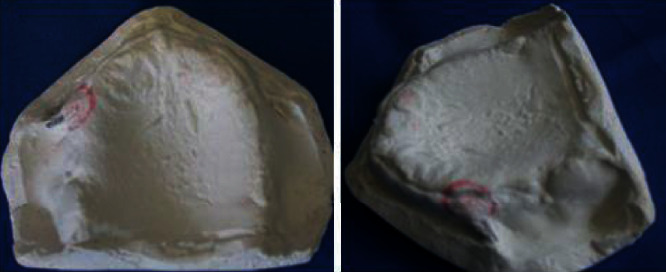
Lateral frenulum scribed on the preliminary cast.

A lateral frenulum on the right maxillary vestibule was noticed and which could impair the retention and stability of the future prosthesis (Figure 25).

Considering this clinical situation and the medical history (bleeding risk), the immediate confection of appropriate new prostheses and surgical removal with laser radiation was proposed and the patient was informed in detail about the surgical procedure.

A habitual procedure for the confection of a complete prosthesis was followed; then, the surgery was proceeded.

The lesion removal was made using a diode laser with a wavelength of 810 nm.

**Figure 26:**
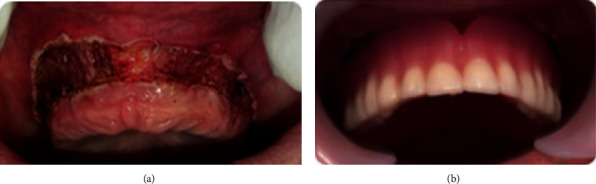
(a) Vestibuloplasty using laser. (b) Surgery control using prosthesis.

.Additionally, a partial vestibuloplasty was performed in the maxillary sulcus and about 3 mm depth extension was gained. The section was extended laterally to eliminate the frenulum (Figure 26(a)).

The treated area was clinically evaluated to verify the absence of bleeding or residual hyperplastic tissue. The maxillary prosthesis served as a guide to evaluate the intervention (Figure 26(b)).

**Figure 27:**
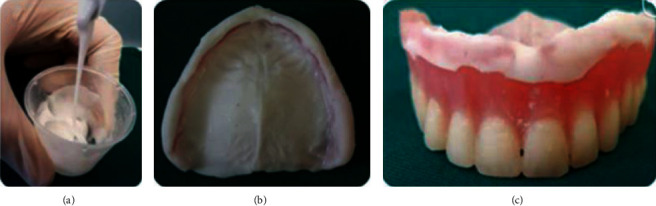
(a) Tissue conditioner consistency. (b) Molded soft tissue conditioner. (c) Soft tissue conditioner border.

Immediately, prostheses were positioned, and the upper one was carefully relined with a soft tissue conditioner (Fitt, Kerr ®) to stabilize the prosthesis and facilitate wound healing (Figures 27(a)–27(c)).

Patient was prescribed with antibiotics and analgesics for 3 days. The postoperative period was asymptomatic, and healing was satisfactory.

**Figure 28:**
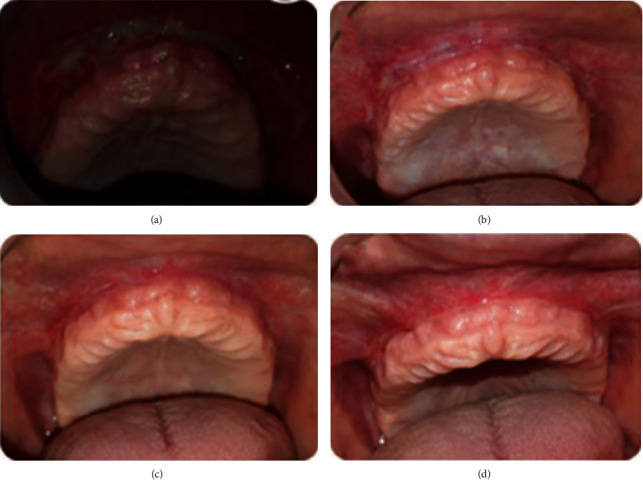
Healing process: (a) 3 days, (b) 1 week, (c) 2 weeks, and (d) 1 month.

The patient was recalled after 3 days, 7 days, and 2 weeks for observation. The healing was good, and no postoperative pain or edema was reported (Figures 28(a)–28(c)).

After 1 month, there was no recurrence of the lesion (Figure 28(d)).

**Figure 29:**
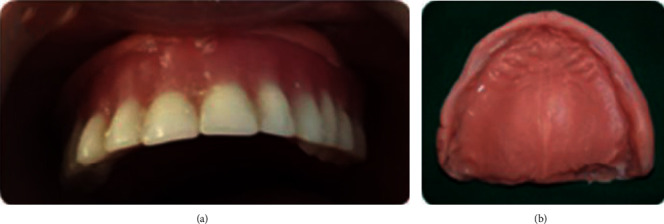
(a, b) Chemopolymerized resin molding.

The patient was satisfied with the prosthesis fit, comfort, and phonetics, so the maxillary prosthesis was rebased with permanent tissue conditioner (DuraBase®) to stabilize the postoperative state (Figures 29(a) and 29(b)).

## 5. Discussion

When supporting tissues are altered, the practitioner must think at first to restore their physiological, histological, and anatomical behavior favorable to a qualitative prosthetic rehabilitation thanks to tissue conditioning. As shown in the three cases, preliminary preparation, defined as all procedures destined to improve the properties of osteomucos-bearing surfaces in contact with complete prosthesis, is indispensable associating the temporarily use of a tissue conditioning, which is the delayed curing acrylic resin and an antifungal agent prescription [4–6, 12–16].

In some cases, when there are no contraindications to surgery, a surgical procedure may be necessary to improve the prognosis of complete prosthesis [17].

Preprosthetic surgery target eliminating certain lesions or abnormalities of the hard and soft tissues of the jaws in edentulous patients such as alveoloplasty, frenectomy, vestibuloplasty, and reduction of hyperplastic mucosa, so that the replacement of the prostheses will be easier and with better prognosis, as it is done to all illustrated patients [15, 17].

According to the stage of the lesion and the general condition of the patient, excision can be performed by either conventional surgical approach that was used in the first and second clinical cases or by laser technique, as we chose to treat the third hypertensive patient.

In fact, since about twenty years, and thanks to a plethora of benefits, laser can be used by dentists, as an effective surgical technique, for the treatment of oral tissue pathologies to improve prosthetic status among elderly. It is a precise and effective treatment modality used to generate both hard and soft tissues and considered an enhancement to a traditional procedure providing a reduced need of anesthesia during surgery and a minimal postoperative edema and pain, making faster the healing process and easier the patient experience.

These advantages are very interesting when treating edentulous elderly patients suffering from systemic health problems that interfere with the therapeutic conduct and the healing process (diabetes, blood hypertension, cardiovascular disease, etc.).

Various types of lasers are used in the surgery of the soft oral tissues such as CO2 laser, ER : YAG laser, ND : YAG laser, diode laser, argon laser, and KTP laser [2, 18–20].

For geriatric patients, the transitional prosthesis is a procedure that can present real advantages and can be recommended with confidence.

Former prostheses may be refitted, vertical dimension corrected, occlusion reconstructed, as well as it being used, after rebasing, as an interim prosthesis, able to serve as a treatment denture until the healing and resorption process of inflamed traumatized tissues is complete, especially in presurgical procedures, as it was illustrated on cases 1 and 2.

On a physiological level, the adaptation is easier with minimal changes for the tongue space, perioral musculature and eating habits [21].

The transitional prosthesis may be used, also, as a postsurgical device to minimize trauma of the operated zone while healing process, to stabilize obtained surgical results and to allow a measured transition to new complete prosthesis status when function and aesthetics are satisfactory, while retaining a good quality of life [21].

When the former prostheses are not suitable for exploitation, the manufacturing of new ones before surgery is indispensable to be used after the preprogrammed intervention. Prosthesis adjustments, either of the occlusal contacts (case 2) or the prosthetic border (case 1), are needed immediately after the surgery to avoid trauma in the area of the wound then reinserted over the surgical bed, after being covered with tissue conditioner in some cases, permitting the maintenance of the new vestibular sulcus depth and width obtained by surgery [22, 23].

An evaluation of the clinical situation of the hard or soft oral tissues is decisive to the prosthetic treatment success. It is the responsibility of the practitioner to establish the right diagnosis and start the coherent treatment plan, as needed, leading to a functional and perfectly integrated complete prosthesis [4, 17].

The outcome of a successful treatment depended not only on the result but also on the way in which it was achieved. Fear and anxiety remain one of the most important barriers for the treatment of the elderly patient, but the transitional denture system can help overcome some of these difficulties.

## 6. Conclusion

Restoring a healthy state of the covering tissues is the first guarantee of a correct registration of the bearing surface, good adaptation of the prosthesis underside surface, and consequently, a satisfactory stability of the complete prostheses.

Management of the bearing surface, by fibromucous tissue cover sanitation, appears as the preliminary decisive step in the therapeutic success leading to functional and perfectly integrated stable complete prosthesis.
